# Who will pay for the malaria vaccines in Africa?

**DOI:** 10.1186/s13690-026-02020-z

**Published:** 2026-07-21

**Authors:** Shuaibu Saidu Musa, Mohamed Mustaf Ahmed, Abdulrahman Garba Jibo, Zhinya Kawa Othman, Don Eliseo Lucero-Prisno

**Affiliations:** 1 School of Global Health, Faculty of Medicine, Chulalongkorn University, Bangkok, Thailand; 2 Department of Nursing Science, Ahmadu Bello University, Zaria, Nigeria; 3 Faculty of Medicine and Health Sciences, SIMAD University, Mogadishu, Somalia; 4 Faculty of Pharmaceutical Sciences, Chulalongkorn University, Bangkok, Thailand; 5 Department of Global Health and Development, Faculty of Public Health and Policy, London School of Hygiene and Tropical Medicine, London, UK; 6 Research Office, Palompon Institute of Technology, Palompon, Leyte Philippines; 7 Office for Research, Extension and Innovations, Bukidnon State University, Malaybalay City, Bukidnon Philippines

**Keywords:** Malaria vaccine, Health financing, Sub-Saharan Africa, Vaccine equity, WHO

## Abstract

Malaria remains one of the most persistent infectious threats in sub-Saharan Africa, where the burden of disease and mortality are disproportionately concentrated among children under five years of age. The World Health Organization’s endorsement of the RTS, S/AS01 vaccine in October 2021 and the R21/Matrix-M vaccine in October 2023 represents a major scientific advance; however, the central question confronting African policymakers, donors, and governments is no longer scientific but financial. In 2024, an estimated 282 million malaria cases and 610,000 deaths occurred worldwide, with the African region accounting for more than 94% of cases and 95% of deaths. The Vaccine Alliance has committed to malaria vaccine introduction through its advance market commitment model; however, its tiered co-financing framework places a fiscal burden on the highest-burden, lowest-income countries. The experience of the COVID-19 pandemic illustrates how vaccine nationalism and concentrated manufacturing can delay equitable access in Africa. Effective vaccine delivery also requires sustained investment in cold-chain systems, the health workforce, and immunisation platforms reaching conflict-affected areas, the costs of which are frequently excluded from procurement estimates. Most African Union member states remain below the Abuja Declaration target of 15% national health spending, sustaining dependence on external financing and undermining health sovereignty. Coordinated action by donors, governments, and manufacturers, including predictable multi-year financing, differential pricing, and measurable domestic allocations, is needed to prevent distribution by purchasing power rather than by epidemiological need. This comment aims to examine who will finance malaria vaccines in Africa and how equitable and sustainable delivery can be secured.

**Table:** 

Text box 1. Contributions to the literature
• Most malaria vaccine literature focuses on clinical efficacy and rollout, leaving the question of who will sustainably finance these vaccines in Africa largely unanswered.
• This comment links the malaria vaccine debate to the wider health financing and vaccine equity literature, framing affordability as a structural rather than a purely scientific challenge.
• It synthesizes prior immunization financing experience, including the Abuja commitment and donor-transition dynamics, to map realistic and equity-driven financing options.
• It offers policymakers and donors concrete entry points, such as predictable multi-year financing, differential pricing, and domestic budget allocations, for sustainable, needs-based delivery.

The WHO’s recommendations for the RTS, S/AS01 malaria vaccine in October 2021 and R21/Matrix-M in October 2023 marked a defining moment in the decades-long fight against one of Africa’s most persistent infectious threats [1, 2]. The two WHO-approved malaria vaccines and their potential to reduce child mortality across sub-Saharan Africa are well documented. However, the central question confronting policymakers, donors, and governments in the entire African region is no longer scientific but financial: who will pay for these vaccines at scale, reliably and over the long term? Without a credible and equitable answer, they risk repeating a familiar pattern in which scientific breakthroughs fail to reach the communities bearing the greatest burden of disease.

In 2024, an estimated 282 million malaria cases and 610,000 deaths were recorded worldwide. The WHO African Region accounted for over 94% of cases and 95% of deaths, of which roughly 75% occurred among children under five years of age [3]. This burden reflects chronic underinvestment in health infrastructure, ecological conditions that favor transmission and health systems struggling to sustain coverage of long-established interventions. Epidemiological progress has stalled in several high-burden countries despite commitments to vector control, seasonal chemoprevention and antimalarial treatment. Additionally, insecticides and drug resistance emergence threatens to erode the remaining gains. Malaria vaccines therefore arrive not at a moment of momentum, but of structural fragility, rendering the financing question a fundamental ethical and political imperative.

The two approved vaccines present significantly different profiles at different study settings and trials. The RTS, S/AS01, developed through a collaboration involving GlaxoSmithKline, the Programme for Appropriate Technology in Health and the Bill and Melinda Gates Foundation; demonstrated 36% efficacy against clinical malaria among children aged 5–17 months who received a four-dose schedule including a booster at month 20, over a median follow-up of 48 months, in a phase 3 trial across 11 centers in seven sub-Saharan African countries. Additionally, efficacy was lower among children who did not receive the booster dose and among young infants aged 6–12 weeks [4]. The, R21/Matrix-M, developed by the University of Oxford and manufactured by the Serum Institute of India, demonstrated 75% efficacy against clinical malaria at sites with highly seasonal transmission and 68% efficacy at sites with perennial transmission, over the 12 months following completion of the three-dose primary series, in a phase 3 trial of children aged 5–36 months; while also carrying a lower projected per-dose cost, offering a logistical advantage for high-volume deployment [5].

Gavi, the Vaccine Alliance, has committed to malaria vaccine introduction through its advance market commitment model [6]. However, the Gavi co-financing framework requires recipient countries to contribute a tiered share of costs, imposing a fiscal burden on nations with the highest malaria mortality rates. Documented inequities in vaccine access and delivery financing raise legitimate questions about the reliability of the current model [7]. A further concern is what happens when countries graduate from Gavi support. Co-financing obligations rise with national income, and many African countries have now entered the accelerated transition phase that leads to full self-financing. Kenya illustrates the strain. It entered accelerated transition in 2022 and is projected to graduate by 2029, with annual vaccine costs expected to climb from about US$10 million to US$38 million [8]. Financial sustainability after graduation is therefore fragile. Late release of co-financing payments has already threatened supply, and uncertainty over vaccine prices complicates affordability during the transition itself. Where domestic budgets cannot absorb the rising share, graduation risks interrupted supply and stockouts. A malaria vaccine added at this point would deepen that exposure unless transition planning is realistic and adequately funded.

The COVID-19 pandemic offers a sobering precedent for the equitable distribution of vaccines in Africa. Although the COVAX facility was established to ensure equitable global access, high-income countries secured bilateral procurement agreements that constrained the supply through multilateral channels. Hence, African countries received vaccines late and in quantities insufficient to achieve meaningful population coverage during the period of greatest epidemiological need [9]. The structural conditions underlying this inequity, including vaccine nationalism and manufacturing concentration in high-income countries remain broadly unresolved. Without deliberate and enforceable policy commitments, there is therefore, no structural guarantee that the distribution of malaria vaccines will follow a different trajectory.

Against this backdrop, Africa is building its own manufacturing response. The African Union and Africa CDC, through the Partnerships for African Vaccine Manufacturing, have set a target for the continent to produce more than 60% of its vaccine needs locally by 2040, up from about 1% currently. To support this, Gavi launched the African Vaccine Manufacturing Accelerator in 2024, a pull-financing mechanism making up to US$1.2 billion available over ten years to help African manufacturers to attain commercial viability, with the first disbursements expected in 2026 [10]. For malaria vaccines, local production offers a long-term financing strategy rather than a short-term fix. It could lower per-dose costs, shorten supply chains, and reduce the exposure to nationalism and donor retreat. The gains depend on aligning these initiatives with predictable demand, pooled procurement, and faster regulatory approval.

It is also noteworthy that, effective malaria vaccine delivery requires reliable cold-chain systems, a well-trained and well-distributed health workforce, and immunisation platforms that reach children in hard-to-reach areas, including conflict-affected areas, yet these delivery costs are frequently omitted from vaccine financing estimates. African governments bear this responsibility. The Abuja Declaration of 2001 committed African Union member states to allocate at least 15% of their national budgets to the health sector; however, more than two decades later, only a small number of countries (about 5.5%) have consistently met that target, and over 30 member states remain well below the 15% benchmark [11]. Development assistance accounts for a substantial share of immunisation spending across many African countries, sustaining dependence on external financing that undermines fiscal resilience and health sovereignty in settings where debt distress constrains domestic expenditure [7]. Approximate figures illustrate the scale of the commitment. On procurement, the R21/Matrix-M price is set to fall to about US$2.99 per dose under a 2025 Gavi and UNICEF agreement, roughly US$12 for the four-dose course, though the Gavi subsidy reduces this to about US$0.20 per dose for the poorest countries [12]. Delivery, cold-chain and health workforce costs then sit on top of the vaccine itself. Peer-reviewed costing of RTS, S introduction estimated total economic programme costs of about US$23 to US$28 per fully vaccinated child, of which the in-country delivery share, net of procurement, added roughly US$2 or more per infant and was driven mainly by labour [13]. These are approximate, setting-specific figures, but they show that non-vaccine costs are substantial and are too often left out of headline financing estimates.

Closing the domestic financing gap will require mechanisms that governments can realistically operate. Several options merit consideration. Earmarked health taxes and social health insurance schemes can broaden the domestic revenue base and pool risk. Sovereign health funds can ring-fence money for immunisation across budget cycles. Innovative instruments, including solidarity levies, airline taxes, and sin taxes on tobacco, alcohol and sugary drinks, can add earmarked revenue. Public-private partnerships can share delivery costs and mobilise domestic capital. However, the evidence urges caution. Revenue from many of these instruments is modest, earmarking is politically and administratively difficult and equity must be protected [14]. These options are therefore complements to higher core budget allocations, not their substitutes.

The scientific breakthrough represented by the two WHO-approved malaria vaccines is substantial, and the moral imperative to deliver their benefits equitably is equally clear (Fig. 1). Donor commitments from Gavi, the World Bank, and bilateral partners must shift from short-term pledges to multi-year, predictable financing that explicitly funds health system strengthening alongside the vaccine itself. African governments must convert declared commitments to health sovereignty into measurable increases in domestic health budget allocations, closing the gap toward the Abuja target rather than restating it. Furthermore, regional pooled procurement, such as the mechanism Africa CDC and UNICEF began operationalising in 2024, should be expanded to aggregate demand across member states, securing more predictable supply and stronger negotiating leverage than fragmented, country-by-country purchasing allows. Also crucial, local manufacturing capacity must scale toward the African Union’s target of producing 60% of the continent’s routine vaccine needs on the continent by 2040, reducing dependence on a handful of external suppliers. Accountability mechanisms including public, comparable reporting of both donor disbursements and domestic budget execution against the Abuja target, are needed so that commitments made are kept. Without progress on all the fronts involved, the malaria vaccine risks becoming yet another advance whose benefits are distributed according to purchasing power rather than epidemiological need, a pattern that African children and the health systems meant to serve them can no longer afford to reexperience.

**Fig. 1:**
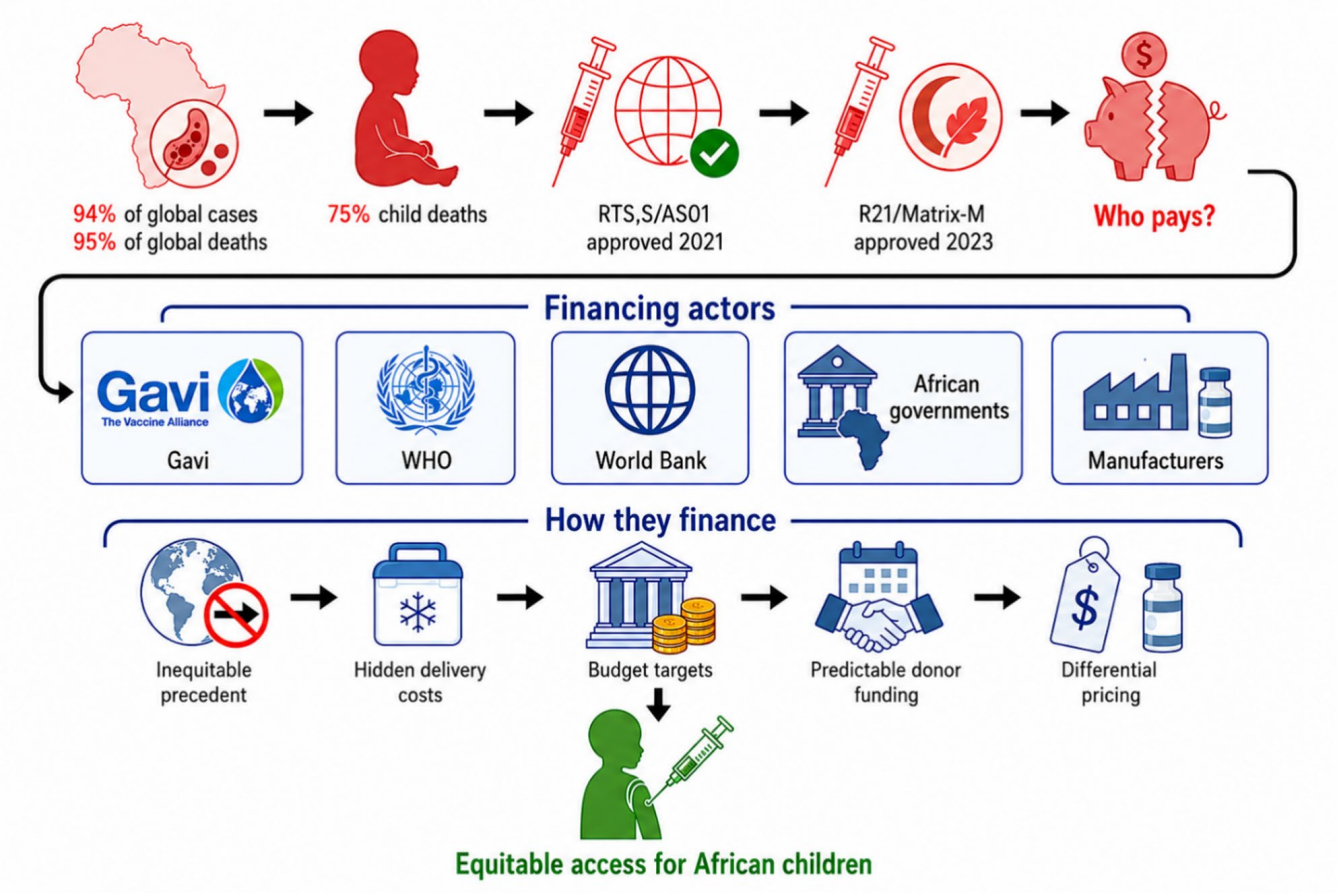
A summary of the malaria burden and vaccine financing in Africa

## Data Availability

No datasets were generated or analysed during the current study.

## Abbreviations

Africa CDC: Africa Centres for Disease Control and Prevention
Gavi: Gavi, the Vaccine Alliance
R21/Matrix-M: R21/Matrix-M malaria vaccine
RTS,S/AS01: RTS, S/AS01 malaria vaccine (Mosquirix)
WHO: World Health Organization
